# Spin Polarization and Quantum Spins in Au Nanoparticles

**DOI:** 10.3390/ijms140917618

**Published:** 2013-08-28

**Authors:** Chi-Yen Li, Sunil K. Karna, Chin-Wei Wang, Wen-Hsien Li

**Affiliations:** Department of Physics and Center for Neutron Beam Applications, National Central University, Jhongli 32001, Taiwan; E-Mails: chiyenli@alumni.ncu.edu.tw (C.-Y.L.); skarna11@alumni.ncu.edu.tw (S.K.K.); 952402004@cc.ncu.edu.tw (C.-W.W.)

**Keywords:** Au nanoparticle, spontaneous particle moment, field induced Zeeman magnetization

## Abstract

The present study focuses on investigating the magnetic properties and the critical particle size for developing sizable spontaneous magnetic moment of bare Au nanoparticles. Seven sets of bare Au nanoparticle assemblies, with diameters from 3.5 to 17.5 nm, were fabricated with the gas condensation method. Line profiles of the X-ray diffraction peaks were used to determine the mean particle diameters and size distributions of the nanoparticle assemblies. The magnetization curves *M*(*H*_a_) reveal Langevin field profiles. Magnetic hysteresis was clearly revealed in the low field regime even at 300 K. Contributions to the magnetization from different size particles in the nanoparticle assemblies were considered when analyzing the *M*(*H*_a_) curves. The results show that the maximum particle moment will appear in 2.4 nm Au particles. A similar result of the maximum saturation magnetization appearing in 2.3 nm Au particles is also concluded through analysis of the dependency of the saturation magnetization *M*_P_ on particle size. The *M*_P_(*d*) curve departs significantly from the 1/*d* dependence, but can be described by a log-normal function. Magnetization can be barely detected for Au particles larger than 27 nm. Magnetic field induced Zeeman magnetization from the quantum confined Kubo gap opening appears in Au nanoparticles smaller than 9.5 nm in diameter.

## 1. Introduction

Metal nanoparticles (NPs) have recently attracted the attention of condensed matter physicists, inorganic chemists and engineers with regard to their fundamental properties and practical applications for the next generation of devices [1,2]. Novel electronic, optical and catalytic properties appear as the size of a bulk material is reduced to the nanometer scale when one encounters the so-called size effects [3–5]. There are three size effects that have been identified which can significantly alter the electron and lattice structures of a particle. Upon reducing the size of a particle to a few hundred of nanometers, the first size effect encountered is the surface effect, where the ratio between the numbers of surface and core atoms is no longer ignorable, so that the surface properties are revealed together with the bulk behavior [6]. Small size effect marks the effect of disruption of the lattice periodicity at the particle surface, where phonon softening and additional low-frequency phonon modes are commonly seen [7–9]. These effects can be expected for particles of a few ten nanometers in diameter. Quantum confinement restricts the spatial motions of electrons and splits the electronic energy bands into discrete narrow sub-bands [10–12]. It governs the electronic behavior of particles smaller than 10 nm in diameter.

Noble metals (Au, Ag, Cu) in their bulk form are known to be weakly diamagnetic. This magnetic property is understood as due to the electron configuration that consists of filled narrow *d*-bands and a single half-filled free electron *s*-band, with the *d*-bands lying far below the Fermi energy, so that the *s-d* hybridization is negligibly small [13]. The filled ion core and *d*-bands give rise to a diamagnetic response to an applied magnetic field, whereas the conduction electrons behave as Pauli paramagnets. In the case of noble metals, the diamagnetic responses are stronger than the Pauli paramagnetic ones, resulting in the diamagnetic character for bulk noble metals. There are at least two effects that may affect the magnetic properties of nano-sized particles. First, the transfer of charges from the surface atoms to the inner ones has been found to be energetically favorable to stabilize the core for nano-sized particles [14,15]. Second, the image charges of the surface electrons, known as Fermi holes [16,17], may result in imbalanced spins near the surface [18]. As a result, the numbers of uncompensated spins increase near the surface, which in turn gives rise to non-zero magnetic moments for the NPs.

Many studies have been performed employing Au NPs for various purposes. Until recently, studies focused on the magnetic properties of the Au NPs themselves are still limited. It, however, begins with the observation of superparamagnetic behavior in 3 nm Au particles embedded in poly *N*-vinyl-2-pyrrolidone [19]. Magnetization measurements made on a series of dodecane-thiol capped Au NPs indicate that the magnetic moment per Au atom is at a maximum in 3 nm particles [20], and magnetic hysteresis is revealed even at room temperature in 1.4 nm particles [21]. Ferromagnetic spin polarization of 1.9 nm Au particles capped by polyallyl-amine-hydrochloride has been identified employing element sensitive X-ray magnetic circular dichronism techniques [22]. Ferromagnetic-like behaviors have also been reported in amine-capped Au NPs, where the magnetic interactions between the Au NPs are noticeably weaker than those in thiol-capped particles, presumably due to the longer organic chains and twinned planes in amine [23]. Intrinsic permanent moments have recently been observed in thiol-capped Au, Ag and Cu NPs at room temperature [24]. Nevertheless, Au NPs stabilized in capping agent of tetraoctyl-ammonium-bromide, which is not covalently bonded but adsorbed on the surface atoms, are found to be diamagnetic [21].

It is clear that the magnetic properties of capped Au NPs can differ significantly from their bulk counterparts. These changes are now understood to originate from the strong chemical affinity of Au atoms to the capping molecules [25,26]. For instance, amine or alcohol groups interact weakly with Au atoms that preserve the electronic properties of the Au in the core, whereas thiols interact strongly with Au atoms that induce noticeable charge redistribution and gives rise to different magnetic properties [27–29]. Amazingly, a great variety of magnetic phenomena, such as giant paramagnetism [30], superparamagnetism [31], and even permanent magnetism [32], have all been observed in thiol-capped Au NPs that have been synthesized using similar chemical routes. The appearance of magnetic moments in thiol-capped Au NPs has been suggested [21] to be associated with the 5*d* localized holes created through the covalent Au-S bonding between the surface Au atoms and the S atoms of the capping thiols. The strong affinity between them can induce a noticeable charge transfer from the surface Au atoms to the S atoms. As a result, the electrons of the surface Au atoms will be redistributed to induce hybridization between the 5*d* and 6*s* electrons, such that the energy of 5*d* electrons is closer to the Fermi level. Consequently, unoccupied densities of *d* states are generated in the surface Au atoms, which make them magnetic [28,33]. An electron transfer of about 0.1 electrons per atom has been estimated [28], which seems to be enough to induce the observed large magnetic anisotropies [21].

Most of the studies made on the magnetic behavior of Au NPs were performed using polymer-capped particles, rarely with bare Au NPs. However, the physical behavior of capped NPs can be significantly altered by the interactions between the Au NPs and the capping agents. In the present article, we aim to discuss the magnetic properties of capping-free Au NPs, with emphasis on the property changes originating from size effects while avoiding the complications that may arise from the capping agents. Our previous studies [34] made on a 4 nm capping-free Au particle assembly reveal paramagnetic, rather than diamagnetic, responses to a driving ac magnetic field. The temperature profile of the average particle moment of a 3.5 nm capping-free Au powder assembly reveal a ferrimagnetic-like spin arrangement, where the moments of the inner and surface atoms point in opposite directions [35].

## 2. Results

### 2.1. Experimental and Theoretical Background

#### 2.1.1. Magnetization Measurement

Magnetization and ac magnetic susceptibility are the physical parameters commonly used to reveal the macroscopic magnetic characteristics of magnetic systems. The former measures the amount of the magnetic moment in the system, whereas the latter picks up the magnetic response of the system to a driving ac magnetic field. In the present studies, the magnetization and ac magnetic susceptibility measurements are performed on a Physical Property Measurement System, manufactured by Quantum Design (San Diego, CA, USA), employing the standard setups. The magnetization M is measured by detecting the induced voltage in the detector coils as the sample moves through them. For ac magnetic susceptibility measurements, the sample is subjected to a weak driving ac magnetic field with the selected frequency and field strength. The responses of the system are detected using two identical sensing coils connected in opposition. Both the in-phase component χ′ and the out-of-phase component χ″ can be measured simultaneously. We note that χ′ measures the response of the system to the driving field, while χ″ reflects the losses of the driving field to the system.

The NPs are very loosely packed for these measurements, so that they reveal mainly the magnetic responses from individual NPs without significant contributions from interparticle interactions. To avoid any aggregation that may arise among the NPs, the powder is shaken at 50 Hz for 5 min using a Vortex-Genie Mixer (Bohemia, NY, USA). The NPs (~50 mg each) are packed into a non-magnetic cylindrical holder provided by Quantum Design (San Diego, CA, USA). The packing fraction *f*, which marks the ratio between the mass densities of the NP assembly in the holder and that of bulk Au, is used to quantify the average interparticle separation that signals the significance of interparticle interactions in the nanoparticle assembly. The packing fraction chosen for all NP powders used in the present studies is ~6%, which corresponds to an average interparticle separation, from edge-to-edge, of 1.25 times the particle diameter. The holder produces a smooth temperature curve and a background signal that is ~2% of the signal from samples.

#### 2.1.2. Langevin Magnetization

**Figure 1:**
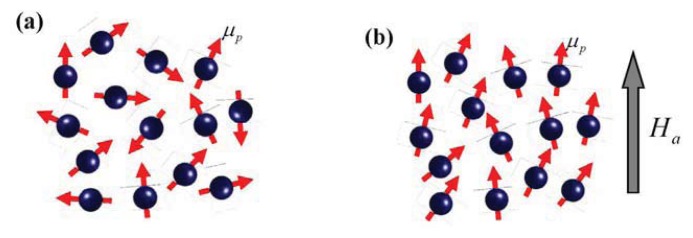
Schematic illustrations of the moment configuration of a nanoparticle assembly. Each carries a particle moment μ_P_ (**a**) without and (**b**) with the presence of an applied magnetic field *H*_a_.

The net magnetization M at a finite temperature for a NP powder consisting of many individual well-separated (interaction-free) NPs, each carrying a net particle magnetic moment *μ⃗_p_* will be zero, when the NPs are naturally packed and the particle moments are randomly oriented, as illustrated in Figure 1a. Under an applied H⃗*_a_* magnetic field, the tendency for the individual *μ⃗_p_* to be aligned along the field direction increases. The magnetic energy takes the form of μ*_p_*λ*H*_a_. The dimensionless permeability λ indicates the net effects from the magnetocrystalline anisotropy, molecular field, and applied magnetic field, such that λ*H*_a_ represents the magnetic field inside the NPs experienced by *μ⃗_p_*. This takes the form of $\lambda =1+(\mu_{0}{\text{M}}_{\text{P}}^{2}/2K)$ for uniaxial spheres [36], where *K* is the energy density associated with magnetocrystalline anisotropy, μ_0_ is the magnetic permeability of free space, and *M*_P_ is the spontaneous saturation magnetization. According to the Boltzmann statistics, the competition between the magnetic interaction energy μ*_p_*λ*H*_a_ and the thermal agitation energy *k_B_*T gives rise to a Langevin profile for the dependency of *M* on *H*_a_ and *T* [37]: M_L_ (H*a*,T) = M_P_ (T)L(*x*) where *M*_P_(*T*) indicates the saturation particle magnetization of the NP powder at a temperature *T*,

$$L(x)\equiv \text{coth}(x)-\frac{1}{x}$$

is the Langevin function with *x* ≡ λ*μp*H*a_/_kB*T, and *k_B_* is the Boltzmann’s constant. The Langevin *M*_L_(*H*_a_,*T*) profile is understood to be a randomly oriented assembly of many interaction-free magnetic NPs with an average particle moment μ*_p_* at a temperature T that are being aligned by the applied magnetic field, as illustrated in Figure 1b.

#### 2.1.3. Zeeman Magnetization

**Figure 2:**
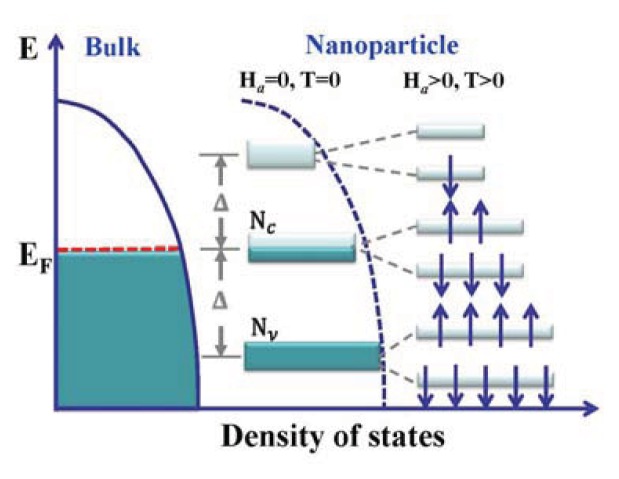
Schematic illustrations of the density of states of bulk Au and the Zeeman split level spectrum near the Fermi level of quantum-confined electrons, assuming an even level separation *Δ* for the discrete spectrum.

When the discrete nature of the electron level spacing becomes visible, magnetization contributed from the condensation of quantum-confined electrons into Zeeman split spin polarized states can also be induced by an applied magnetic field. This *H*_a_-induced magnetization is mainly contributed from the conduction electrons. A schematic plot of the Zeeman split level spectrum near the Fermi level for quantum-confined electrons is shown in Figure 2, assuming that the level separations of the discrete spectrum take the same value of *Δ*. At a finite temperature, thermal excitations from conduction electrons into the first excited band and from the valence electrons into the conduction band are equally probable. It is then the available density-of-states for excitations measure the contributions to the Zeeman magnetism. The induced magnetization of these quantum spins follows a Brillouin profile [37]:, where M_Z_ represents the induced saturation magnetization,

$$B_{\text{J}}(y)\equiv \frac{2\text{J}+1}{2\text{J}}\text{ctnh}\left[\frac{(2\text{J}+1)y}{2\text{J}}\right]-\frac{1}{2\text{J}}\text{ctnh}\left(\frac{y}{2\text{J}}\right)$$

is the Brillouin function of order *J*, *y* = *g*μ_B_λ*H*_a_/*k*_B_*T*, *g* is the Lande *g*-factor, and μ_B_ is the Bohr magneton. The Brillouin function reduces to a Langevin function when *J* = ∞ that is *B*_∞_(*y*) = *L*(*y*), and it reduces to a hyperbolic tangent function when *J* = 1/2 that is *B*_1/2_ = tanh(*y*). Competition between thermal agitation and field alignment results in a Langevin type of magnetization curve *M*_L_(*H*_a_, *T*), whereas the thermal excitation of the valence and conduction electrons into Zeeman split spin polarized states gives rise to a Brillouin magnetization *M*_B_(*H*_a_,*T*). Although the thermal profile of the Brillouin function is different from that of the Langevin function, they look similar.

#### 2.1.4. Size Dispersion

Detecting the ultimate but extremely weak magnetic signals from a single NP is hardly achievable. The magnetic signals can be greatly enhanced by using a collection of NPs, but mono-dispersed powder is difficult to find. It has been shown that ignoring the influences from size dispersions of NP powders can result in unphysical conclusions [38]. In considering the magnetization from a NP powder, the contributions from particles of different sizes must be taken into account. The Langevin magnetization then takes the form of

(1) $${\text{M}}_{\text{L}}({\text{H}}_{a},\text{T})=\sum_{i}n_{i}\mu_{pi}L\left(\frac{\mu_{pi}\lambda_{i}{\text{H}}_{a}}{k_{B}\text{T}}\right)=\sum_{i}n_{i}\mu_{pi}L\left(\frac{\mu_{pi}\lambda {\text{H}}_{a}}{k_{B}\text{T}}\right)$$

where *n_i_* is the number of particles with a particle moment μ*_pi_*, λ*_i_* is the magnetic permeability of the corresponding particle that can be taken as a constant value λ for a narrow-dispersed powder. It is unphysical to extract all the parameters associated with the above expression from the observed *M*_L_(*H*_a_,*T*) unless the number of free parameters can be largely reduced. A log-normal size distribution is often detected for a powder consisting of quantum NPs, so that

(2) $$n_{i}(d_{i})=\frac{1}{d_{i}\sqrt{2\pi }\sigma }\text{exp}\left\{-\frac{{\left(\text{ln }d_{i}-\text{ln }d_{m}\right)}^{2}}{2\sigma^{2}}\right\}$$

where *d_m_* represents the mean particle diameter of the powder and *σ* is the standard deviation of the distribution. In addition, assuming that the particle moment reaches a maximum value at a finite size and its dependency on the particle size can be described by an analytical expression, the μ*_pi_*(*d_i_*) can then be expressed into three parameters of the maximum particle moment μ*_pm_*, the mean particle diameter *d_c_*, and the standard deviation *w*. In the cases of the log-normal type of size dependency, the particle moment becomes

(3) $$\mu_{pi}(d_{i})=\frac{\mu_{pm}}{d_{i}\sqrt{2\pi }w}\text{exp}\left\{-\frac{{\text{(ln }d_{i}-\text{ln }d_{c})}^{2}}{2w^{2}}\right\}$$

A plot of *n_i_*μ*_pi_* against *d_i_* using the parameters obtained from the fit reveals the contribution from each size of particles to the saturation magnetization of the NP assembly.

### 2.2. Intrinsic Magnetic Moment

#### 2.2.1. From Diamagnetic to Superparamagnetic Responses

**Figure 3:**
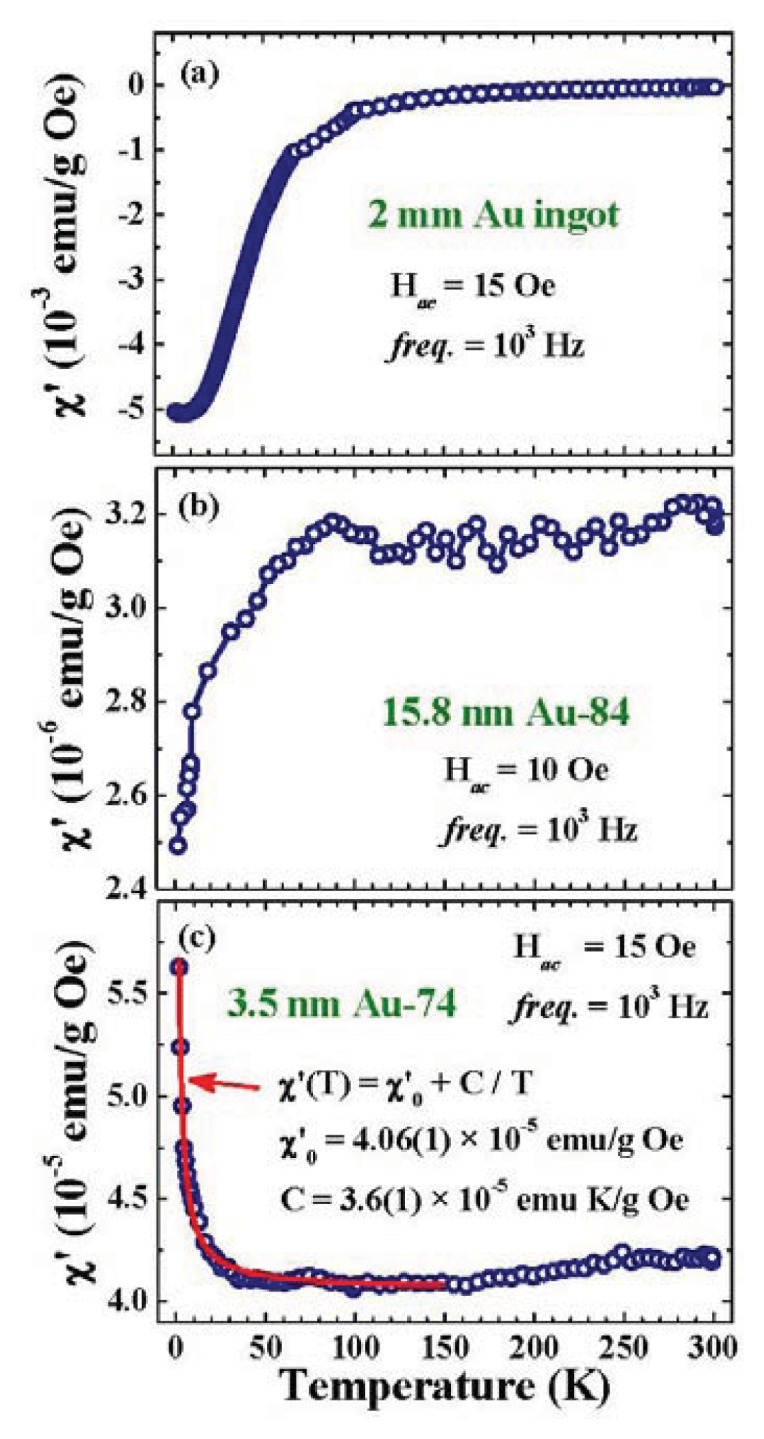
Temperature dependencies of the in-phase component χ′ of the ac magnetic susceptibility of (**a**) 2 mm Au spheres; (**b**) 15.8 nm Au-84 and (**c**) 3.5 nm Au-74 particle assemblies, measured employing a driving ac magnetic field with a root-mean-square strength of 15 Oe and a frequency of 10^3^ Hz. The solid curve in (**c**) indicates the results of fits for the Curie-Weiss thermal profile.

It is known that the Lenz diamagnetic response from the filled *d*-band is larger than that of the Pauli paramagnetic response from the half-filled *s*-band, which results in a diamagnetic characteristic for noble metals in their bulk form. This situation can be altered as the size of the system is reduced to the nanometer scale, where charge transfer and Fermi-hole effects are significant. Figure 3 shows the thermal profiles χ′ (*T*) of the 3.5 nm Au-74, 15.8 nm Au-84 assemblies, and 2 mm spheres (representing bulk Au) for a direct comparison. Diamagnetic responses are seen for 2 mm ingots at all temperatures studied (Figure 3a), as expected. However, noticeable reductions in the diamagnetic responses are revealed upon warming, with a thermal reduction rate that is significantly reduced by ~100 K. This behavior cannot be a result of the increases of Pauli paramagnetism at high temperatures, but instead must be a result of the decreases of Lenz diamagnetism. Lenz diamagnetism is known to be associated with the tendency of core electrons to shield the interior of the system from the external magnetic field. Its magnitude is proportional to the mean square of the perpendicular distance of the shielding electrons from the field axis through the nucleus [39]. The smaller diamagnetic responses observed at higher temperatures reflect that thermal excitations can significantly the diamagnetic response.

Interestingly, the 15.8 nm Au-84 assembly exhibits paramagnetic responses at all temperatures studied (Figure 3b), showing that the diamagnetic signals from the filled *d*-band have been overcome by the paramagnetic responses. However, the stronger diamagnetic responses at lower temperatures are still evident, so that the net paramagnetic signals are smaller at lower temperatures. Curie-Weiss-like superparamagnetic responses are seen in the 3.5 nm Au-74 assembly (Figure 3c). These χ′ were collected without the presence of *H*_a_, but reveal the responses of the particles to the driving magnetic field. One way to understand this behavior is that the Pauli paramagnetic responses are greatly enhanced in nano-sized particles, so that they overcome the weakened diamagnetic ones. It is known that the Pauli paramagnetic response is proportional to the density of states at the Fermi energy *g*(*ɛ*_F_). The largely enhanced paramagnetic responses will require that *g*(*ɛ*_F_) is also greatly increased, if there is no spontaneous magnetic component. Although *g*(*ɛ*_F_) can be altered or even enhanced in nano-sized particles, it still hardly accounts for the three orders-of-magnitude enlargement in the Pauli paramagnetic responses. The existence of spontaneous magnetic moments in nano-sized Au particles is needed to understand the thermal characteristics of χ′.

#### 2.2.2. Spontaneous Particle Moment

**Figure 4:**
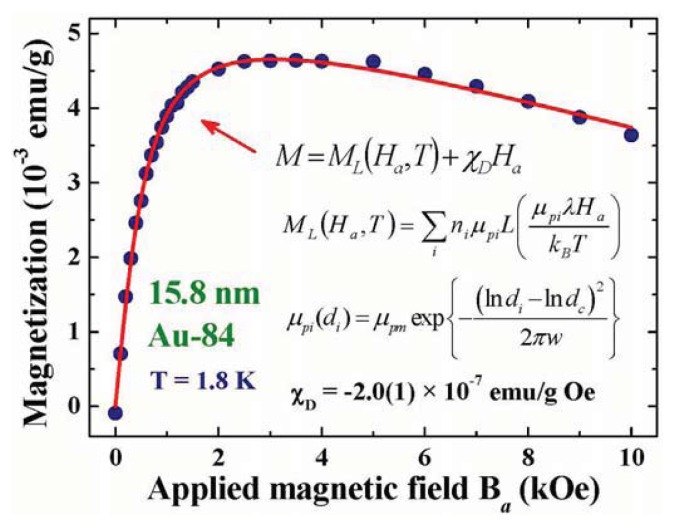
Field profile of the magnetization of 15.8 nm Au-84 particle assembly at 1.8 K. The solid curve indicates the results of fits for a Langevin paramagnetic plus a Lenz diamagnetic component, assuming a log-normal dependency of the particle moment on diameter.

**Figure 5:**
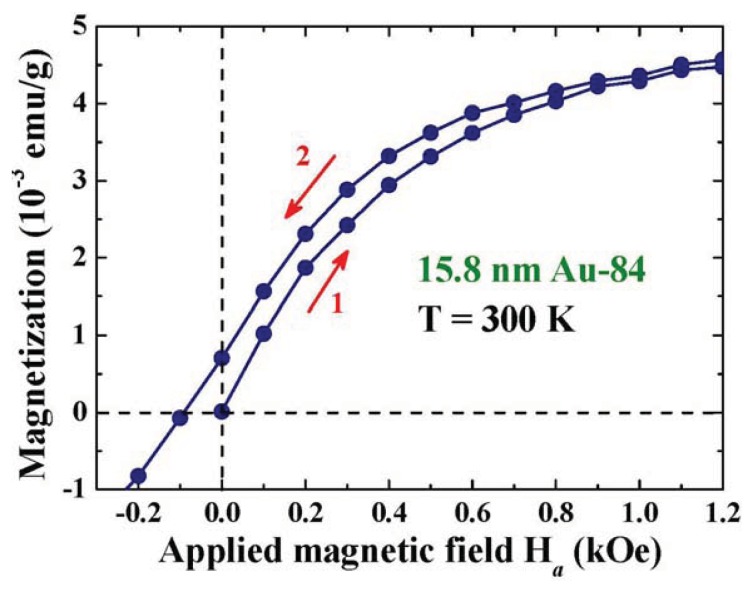
Magnetic hysteresis loops of the 15.8 nm Au-84 assembly at 300 K, showing a coercive field of 100 Oe and a romance of 7 × 10^−4^ emu/g at 300 K.

Magnetization is a physical quantity that measures the net magnetic moment of the system. Magnetization measurement made on a multi-domain system is frequently performed with the presence of an *H*_a_, which serves to align the magnetic moments of the domains along the field direction. The differences between the measurements made in a field-increasing loop and in a field-decreasing loop can reflect the character of the spontaneous magnetic moment of the system. Figure 4 illustrates the *M*(*H*_a_) curves of the 15.8 nm Au-84 assembly at 1.8 K, taken in a field-increasing loop. In the low *H*_a_ regime, the magnetization increases rapidly with increasing *H*_a_, becoming saturated at *H*_a_ ≈ 2 kOe. This *M*(*H*_a_) curve may be satisfactorily described by a Langevin profile, which signifies the existence of ferromagnetic spin correlations in the 15.8 nm Au-84 assembly. The observed Langevin *M*(*H*_a_,*T*) curves may be understood as the magnetic moments of the randomly oriented assembly of non-interacting magnetic Au NPs of average particle moment μ_p_ at a temperature T that are being aligned by the applied magnetic field *H*_a_. Lenz diamagnetic responses are revealed in the high *H*_a_ regime, such that the magnetization decreases linearly with increasing *H*_a_. Similar *M*(*H*_a_) profiles are also observed with temperatures up to 300 K. The existence of spontaneous particle moments in Au NPs is also evident in the field-increasing and field-decreasing magnetization loops. Magnetic hysteresis is clearly revealed in the low *H*_a_ regime of the *M*(*H*_a_) loops even at 300 K, as shown in Figure 5. A coercive field of 100 Oe and a remanence of 7 × 10^−4^ emu/g for the 15.8 nm Au-84 assembly at 300 K are clearly seen. Although the coercive field and remanence are relatively small, they are nevertheless direct evidences of the existence of a spontaneous magnetic moment in the 15.8 nm Au-84 assembly.

**Figure 6:**
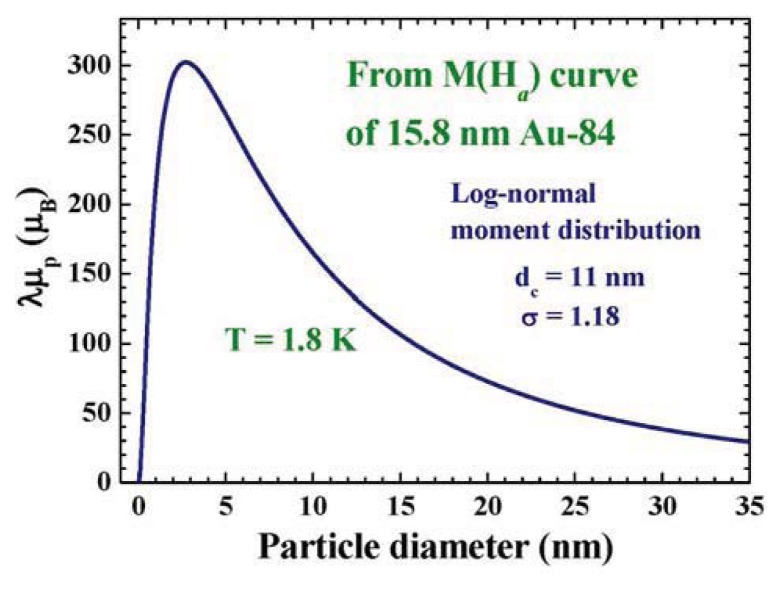
Size dependence of λμ*_p_* obtained from the *M*(*H*_a_) curve of the 15.8 nm Au-84 particle assembly at 1.8 K, where λ is the dimensionless permeability and μ*_p_* is the average particle moment. A λμ*_p_* = 67 μ_B_ for the 15.8 nm Au particle at 1.8 K is obtained.

Two magnetic components are observed: a paramagnetic component that may be described using the Langevin profile, plus a diamagnetic one that responds linearly to *H*_a_. A mono-dispersed powder sample is not available, therefore, the nature of the size dispersion must be accounted for when analyzing the *M*(*H*_a_) curve measured on a powder sample. The solid line in Figure 4 indicates the results of the fits for *M*(*Ha*) = *M*_P_*L*(*x*) + χ_D_*Ha*, obtained by knowing the size distribution of the 15.8 nm Au-84 assembly (Table 1) and assuming a log-normal type of particle moment dependency on the particle size (equation 3), with λμ*_pm_*, *d_c_*, *w*, and χ*_D_* being the fitting parameters. Unfortunately, the permeability λ and maximum particle moment μ*_pm_* cannot be separated in this fit, since they are coupled parameters in the Langevin expression. Good agreement between the observations and the fit is obtained. The size dependence of the particle moment obtained from the fits is shown in Figure 6, which gives a λμ*_p_* = 100 μ_B_ for the 15.8 nm Au-84 assembly at 1.8 K. We note that λμ*_p_* of other sizes can also be extracted from the analysis, which is a direct consequence of the assumption made that the variations of particle moment with particle diameter can be expressed by an analytical function. We remark that the *M*(*H*_a_) curves fit equally well to a Langevin profile without considering the size dispersed nature of the powder, but gives a value of λμ*_p_* = 83 μ_B_ at 1.8 K for the assembly. This represents the averaged λμ*_p_* of the assembly, but ignores the uneven nature of number fractions. The μ*_p_* obtained when ignoring the size dispersion is 17% smaller. It represents the particle moment of a specific size significantly less precisely.

#### 2.2.3. Quantum Spins

**Figure 7:**
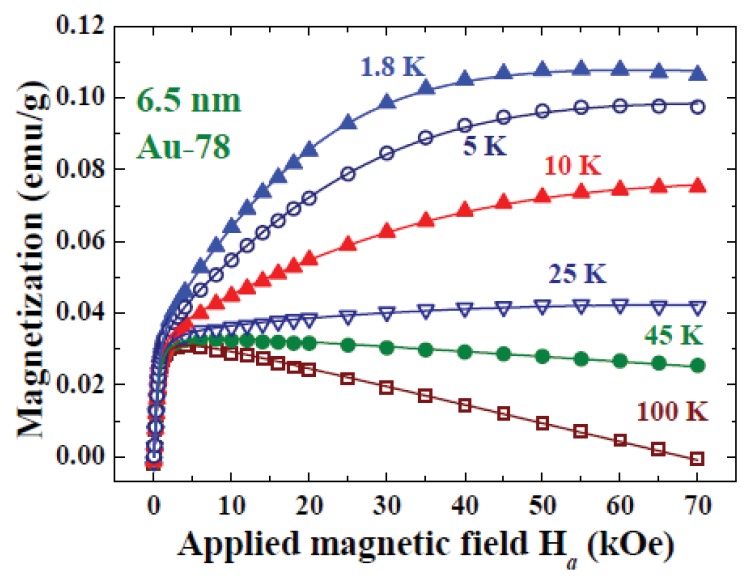
Magnetization curves of the 6.5 nm Au-78 particle assembly, taken in field-increasing loops at six representative temperatures. The Langevin and Lenz components are clearly revealed in the *M*(*H*_a_) curves taken above 25 K. An additional Brillouin component appears in the high *H*_a_ regime of the *M*(*H*_a_) curves taken below. The solid curves indicate the fits to a Langevin plus a Lenz plus a Brillouin profile of order *J* = 1/2.

Quantum confinement causes the electronic bands to split into discrete sub-bands separated by Kubo gaps. An additional contribution to the magnetization from the *H*_a_-induced spin polarization will appear in the *M*(*H*_a_) curves taken at low temperatures, where thermal excitations through the Kubo gap are not severe. Figure 7 displays the *M*(*H*_a_) curves of the 6.5 nm Au-78 assembly, taken at six representative temperatures and in a field-increasing loop. Langevin and Lenz contributions to M are clearly revealed in the *M*(*H*_a_) curves taken at 100 and 45 K. An additional component is clearly revealed in the high-*H*_a_ regime of the *M*(*H*_a_) curves taken at lower temperatures. This Zeeman component is hard to see at 25 K, showing that the thermal populations from the down-spin state to the up-spin state have nearly decompensated for the *H*_a_-induced magnetization. Assuming that there is no significant coupling or cross-talking between the Langevin and Zeeman components, the *M*(*H*_a_) curves can then be expressed as additive compositions of a Langevin, a Brillouin component and a Lenz component. This assumption can be justified as the moment-alignment Langevin signals have saturated at a field far below where the *H*_a_-induced Brillouin signals noticeably developed. The solid lines in Figure 7 indicate the results of the fits for *M*(*Ha*) = *M*_P_*L*(*x*) + *M*_Z_*B*_J_(*y*) + χ_D_*Ha*, with a Brillouin function of order *J* = 1/2 that disappears above 45 K. Fits assuming *J* ≥ 0.86 give a nonphysical λ < 1. A *J* = 1/2 signals that only the spin magnetic moment contributes to the induced Zeeman magnetization. This result agrees with that the Kubo bands in Au NP are mainly associated with the delocalized conduction electrons. In addition, the permeability λ and maximum particle moment μ*_pm_* can be separated in the fits, since they are not coupled parameters in the Brillouin expression. The value λ = 1.16 is obtained from the *M*(*H*_a_) curve at 1.8 K.

**Figure 8:**
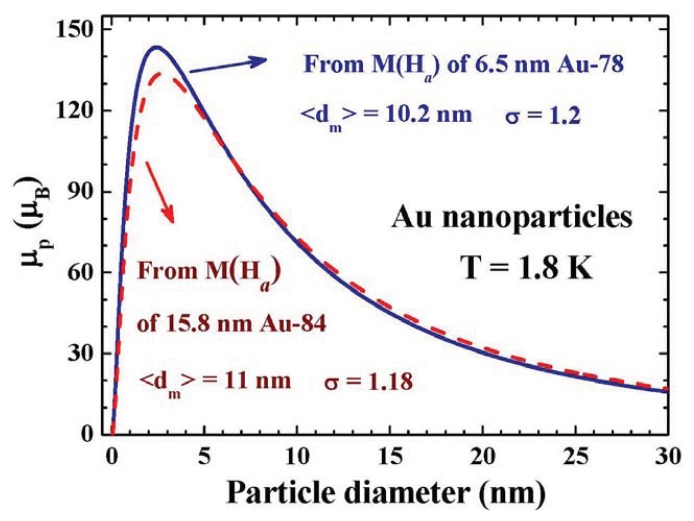
Direct comparison between the size dependence of μ*_p_* obtained from the *M*(*H*_a_) curves of the 6.5 nm Au-78 (solid curve) and 15.8 nm Au-84 (dashed curve) particle assembly at 1.8 K. The two μ*_p_*(*d*) curves match very well, revealing that the maximum particle moment will appear in the 2.40 nm particles.

It is remarkable to find that the size dependencies of the particle moment obtained from the 15.8 nm Au-84 and 6.5 nm Au-78 assemblies agree very well. A direct comparison can be made by taking λ = 2.26 for the 15.8 nm Au-84 assembly, as shown in Figure 8. Accordingly, the maximum particle moment will appear in the 2.4 nm particles, with μ*_p_* = 143 μ_B_ at 1.8 K, for the face-centered cubic (fcc) Au NPs. The particle moment will be reduced to below 10 μ_B_ in the 40 nm Au particles, which is hardly detectable using conventional means. There are 424 Au atoms in a spherical 2.4 nm fcc Au particle. We can then expect a magnetic moment of 143/424 = 0.34 μ_B_ for the Au ions in the 2.4 nm NP, when assuming the magnetic moments are evenly distributed in the NP and the thermo-induced moment [40,41] plays no significant effect at 1.8 K. We note that ultra-small NPs may crystallize into a Mackay icosahedral structure [42], rather than an fcc structure. The Mackey icosahedral structure can generate a larger particle moment from the higher coordination number and lower structural symmetry of the icosahedral packing [35].

**Figure 9:**
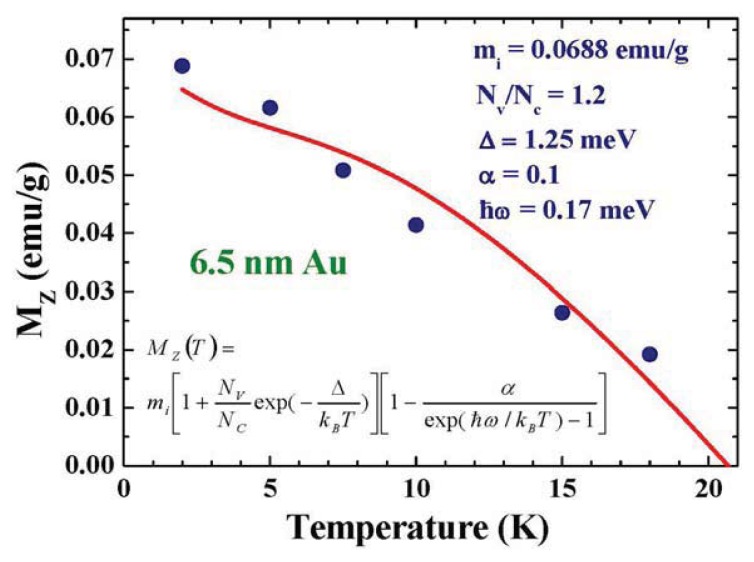
Temperature dependence of the induced saturation magnetization M_Z_ of the 6.5 nm Au-78 assembly. The solid line indicates the results of fits for the expression listed in the plot, giving an average level separation of *Δ* = 3.13 meV.

The variations of M_Z_ with temperature are shown in Figure 9. Thermal reductions of M_Z_ are linked to the creations of thermal magnons. The noticeable drops of M_Z_ upon warming at low temperatures indicate a small energy for the associated thermal magnons. In addition, the valence electrons can also contribute to M_Z_ at finite temperatures, as shown in Figure 4(2). This takes the form of *N*_v_μ_B_ exp (−Δ/*k*_B_*T*) *B*_J_(*y*), where *N*_V_ is the total number of valence electrons. Accounting for the contributions from the conduction and valence electrons and taking the thermal fluctuations of M_Z_ as being proportional to the number of thermal magnons, the induced magnetization takes the form of

(4) $${\text{M}}_{\text{Z}}=m_{i}\left[1+\frac{{\text{N}}_{\text{V}}}{{\text{N}}_{\text{C}}}\text{exp}\left(-\frac{\Delta }{k_{B}T}\right)\right]\;\left[1-\frac{\alpha }{\text{exp}(\hbar \omega /k_{B}T)-1}\right]$$

where N_C_ is the total number of conduction electrons, Δ is the Kubo gap, *α* is the normalization factor, ħω is the energy of the thermal magnon, and m*_i_* is the proportional constant. The solid curve in Figure 9 indicates the fitted curve for Equation 4, with the parameters listed in the plot. Accordingly, M_Z_ disappears at 21 K, which indicates the temperature at which the thermal populations from the down-spin states onto the up-spin states have decompensated for the Zeeman magnetization induced by *H*_a_. The value of *Δ* = 1.25 meV obtained from the fit is noticeably larger than the expected value of 0.76 meV, when estimated using the Kubo formula and taking the same electron density as for the bulk Au for nanoparticles [12]. The larger observed Kubo gap can be a direct result of the electron density at the Fermi surface of Au nanoparticles which is indeed smaller than that of bulk Au.

### 2.3. Size Dependence

#### 2.3.1. Critical Particle Size

**Figure 10:**
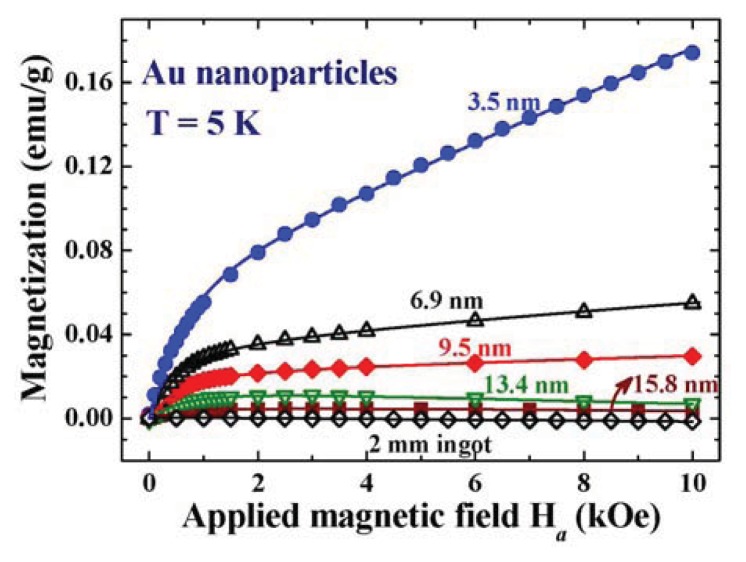
*M*(*H*_a_) curves of five representative Au nanoparticle assemblies together with that of 2 mm Au spheres at 5 K. The Zeeman magnetization appears in the 9.5 nm Au-80 and smaller particle assemblies, but is absent in the 13.4 nm Au-82 and larger particle assemblies.

Both the Lagnevin and Brillouin magnetizations depend strongly on the particle size. The critical particle size for the appearance of Zeeman quantum spins can be estimated using the Kubo formulism. There are, on the other hand, no theoretical bases available for estimating the critical particle size for the development of spontaneous magnetic moments in Au nanoparticles, instead we rely on experimental information. The *M*(*H*_a_) curves of five representative Au nanoparticle assemblies together with that of 2 mm Au ingots at 5 and 300 K are shown in Figures 12 and 13, respectively. Lenz diamagnetic responses, with magnetic susceptibilities of −1.9(1) × 10^−7^ and −2.1(1) × 10^−7^ emu/g Oe at 5 K and 300 K, respectively, are revealed in the 2 mm Au ingots. Diamagnetic susceptibilities of −2.3 × 10^−7^ and −2.6 × 10^−7^ emu/g Oe at 5 and 300 K, respectively, were obtained for the 15.8 nm Au-84 assembly, which are ~20% higher than the corresponding values for the 2 mm ingots. Zeeman magnetization is absent in the 13.4 nm Au-82 and larger particle assemblies at 5 K (Figure 10), showing that the energy gaps between the Kubo sub-bands are insignificant in the 13.4 nm Au particles at 5 K. A Kubo gap of *Δ* = 0.087 meV is expected for the 13.4 nm Au particles, when estimated using the Kubo formula and assuming the same electron density as for bulk Au for the nanoparticles. The thermal energy at 5 K is indeed enough to equally populate the up-spin and down-spin states, which smear the appearance of relatively small Kubo gap.

**Figure 11:**
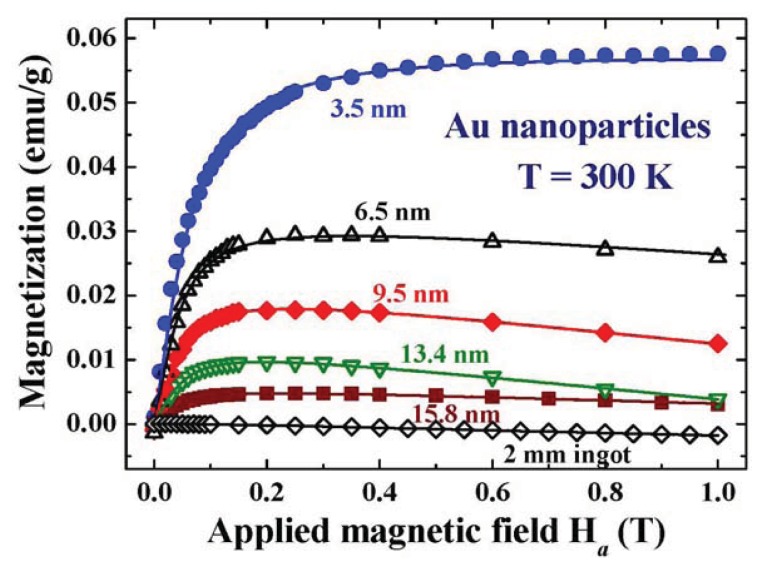
*M*(*H*_a_) curves of five representative Au nanoparticle assemblies together with that of 2 mm Au spheres at 300 K. Spontaneous magnetization is clearly revealed even in the 15.8 nm Au-84 at 300 K.

**Figure 12:**
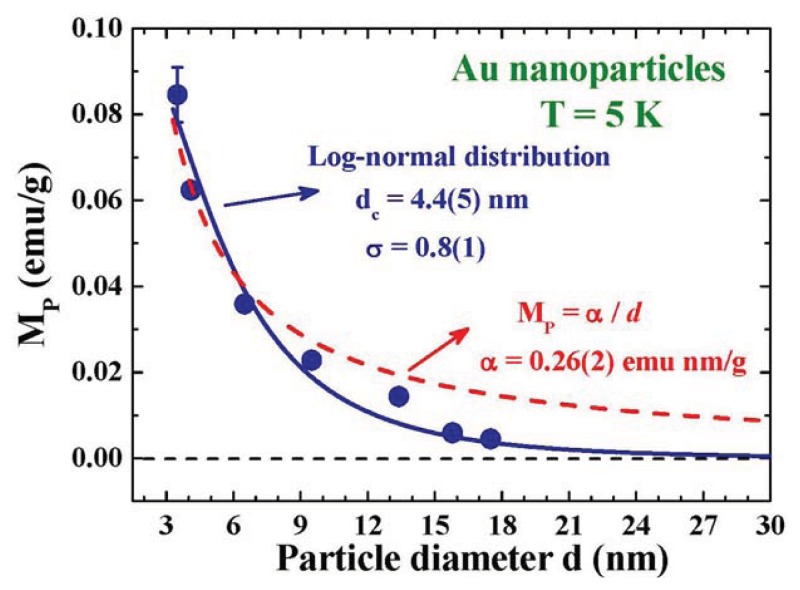
Size dependence of the saturation spontaneous magnetization *M*_P_ at 5 K. It departs significantly (dashed curve) from the 1/*d* dependence, but can be described by a log-normal profile (solid curve) with a center at 4.9 nm and a standard deviation width of 0.81.

The spontaneous magnetization is clearly revealed even in the 15.8 nm Au-84 at 300 K (Figure 11). The critical particle diameter for the appearance of spontaneous magnetic moment in Au nanoparticles can be revealed by examining the dependency of *M*_P_ on particle size, as shown in Figure 12. *M*_P_ increases with decreasing particle size. It reaches 0.085 emu/g for the 3.5 nm Au-74 assembly. The *M*_P_ of an assembly can be calculated by the convolution of the μ*_p_*(*d*), as shown in Figure 8 and the *n*(*d*) of the assembly, as listed in Table 1. A value of *M*_P_ = 0.09 emu/g is obtained for the 3.5 nm Au-74 assembly, which matches very well with the observed 0.085 emu/g. The *M*_P_ is linked to the amounts of available unpaired spins, which connect to the numbers of atoms on the surface of the particles in the assembly. The fact that a smaller particle will develop a larger magnetic moment is qualitatively understandable. A *M*_P_~1/*d* will be expected, when assuming the numbers of surface atoms that are available for transferring charges into the core is the only mechanism that is operating in the development of magnetic moments in nanoparticles. The observed *M*_P_(*d*) departs (dashed curve in Figure 12) significantly from the 1/*d* dependence, showing that mechanisms other than the numbers of surface atoms play important roles as well. Interestingly, the *M*_P_(*d*) curve can be described by a log-normal distribution function (solid curve in Figure 12) with a maximum at 2.3 nm, which agree reasonably well with the 2.4 nm obtained from the *M*(*H*_a_) analysis shown in Figure 8. The observed log-normal *M*_P_(*d*) profile also agrees with the assumption made on the log-normal dependency of the particle moment on size in the *M*(*H*_a_) analysis. *M*_P_ reduces to 10% and then 1% of the maximum value in particles larger than 13 and 27 nm, respectively, where spontaneous magnetization becomes barely visible even if it does exist.

#### 2.3.2. Thermal Excitations of Surface and Core Moments

**Figure 13:**
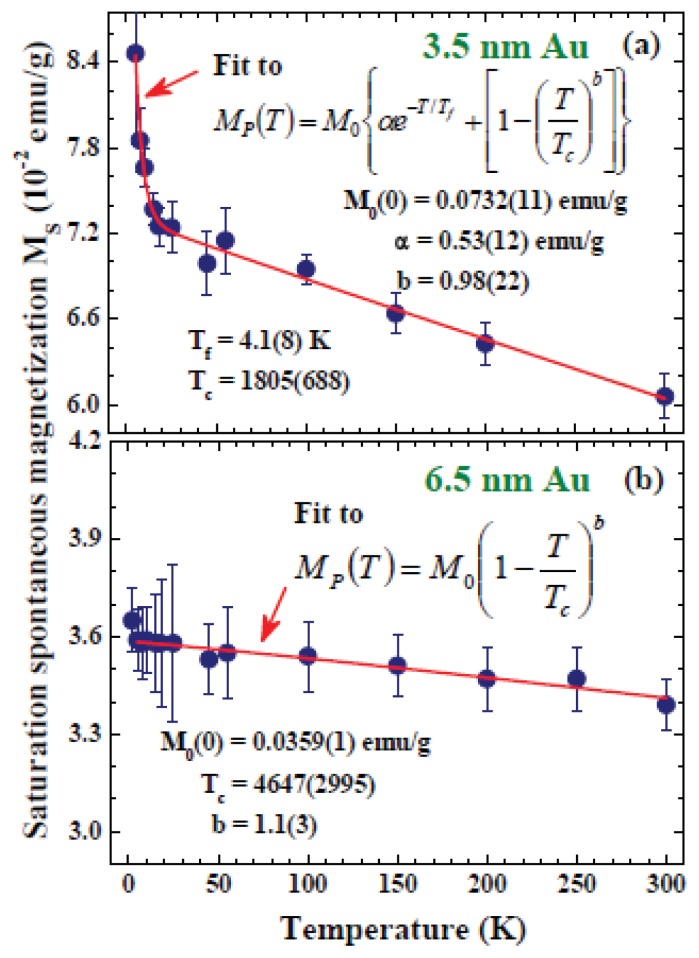
Temperature dependencies of the saturation spontaneous magnetization of (**a**) 3.5 nm Au-74 and (**b**) 6.5 nm Au-78 particle assemblies. The solid curves indicate the results of fits for the expression listed in the plot.

One of the most extraordinary parts of the nature of nanoparticles is that the surface and core characteristics can both be revealed. It can be expected that the magnetic moments developed on the surface of a nanoparticle are different from those developed in the core. The spontaneous magnetization *M*_P_ contains the contributions from both surface and core moments, and their thermal characteristics can be noticeably different. The temperature dependence of *M*_P_ of the 3.5 nm Au-74 and 6.5 nm Au-78 assemblies can be seen in Figure 13a,b, respectively. The temperature profiles of *M*_P_ for Au NPs are fundamentally different from what is expected for a magnetization reduction due to the creation of coherent magnons, where a Bloch T_3/2_ law is anticipated for bulk ferromagnets [43]. The *M*_P_ of 3.5 nm Au-74 decreases rapidly with increasing temperature, but the thermal reduction rate is largely reduced above 25 K. A 15% decrease in *M*_P_, seen from 25 to 300 K, demonstrates that the transition temperature is significantly higher than 300 K. The large difference observed for the thermal reduction rates below and above 25 K, shows that they are linked to different origins. An upturn in the thermal profile of the saturation magnetization has also been observed in NiFe_2_O_4_ ferrite nanoparticles embedded in a SiO_2_ matrix [44], which is attributed to the creation of spin-wave excitations that appear in the finite-sized nanoparticle [45]. In the present case of bare Au nanoparticles, this indicates separate contributions from the surface spins and from the core spins to the saturation magnetization. It is likely that the surface moments could become disordered at a lower temperature and with a higher thermal reduction rate. The sharp turn at 25 K separates the effects mainly resulting from the surface moments at low temperatures and from the core moments at high temperatures.

A characteristic temperature, known as the surface spin freezing temperature *T*_f_, has been proposed [46,47] to scale the thermal agitations of surface moments into an exponential reduction profile of *M*_P_ proportional to exp(−*T*/*T*_f_). On the other hand, the thermal agitation of the core spins can be described by a power law of *M*_P_ proportional to [1 − (*T*/*T*_C_)*^b^*], where T_C_ is the transition temperature and the exponent *b* links to the type of spin ordering [43]. Accounting for the contributions from surface spins as well as from the core spins, the saturation magnetization can be expressed as

(5) $${\text{M}}_{\text{P}}\;(\text{T})={\text{M}}_{0}\left\{\alpha e^{-\text{T}/{\text{T}}_{f}}+\left[1+{\left(\frac{\text{T}}{{\text{T}}_{C}}\right)}^{b}\right]\right\}$$

where *M*_0_ is the saturation magnetization of the core moment at zero temperature and *α* specifies the ratio between the surface and core moments at zero temperature. In this expression the saturation magnetization at zero temperature is *M*_P0_ = *M*_0_(*α* + 1). The solid curve in Figure 13a indicates the results of fits for Equation 5, giving *M*_P0_ = 0.073 emu/g, *α* = 0.53, *T*_f_ = 4.1 K and *b* = 0.98. This value of *b* = 0.98 obtained for the 3.5 nm Au-74 is significantly smaller than the *b* = 3/2 expected for creations of thermal magnons in bulk ferromagnets [43] and the *b* = 2 expected for finite-size ferromagnetic clusters based on a mean field calculation [48]. Modification of Bloch T_3/2_ law has also been observed in Mn_0.6_Fe_0.4_Fe_2_O_4_ nanoparticles that emerged in a ferrofluid [49]. The *α* = 0.53 obtained from the fits showing that the surface moment is 53% of the core moment in 3.5 nm Au-74.

The *M*_P_(*T*) of 6.5 nm Au-78 assembly decreases monotonically with decreasing temperature, where the contributions from the surface spins and from the core spins are not separately revealed. This can be the result of a considerably smaller T*_f_* or a significantly smaller thermal reduction rate of the surface moment for the 6.5 nm Au-78 assembly. A smaller T*_f_* means the surface spins will be disordered at a lower temperature (below 1.8 K for Au-78), which is hardly the case, since the core spin moment is still clearly visible at 300 K. It is very unlikely that the ordering temperatures of the surface moment and core moment will differ by two orders of magnitude. A flat thermal reduction rate observed in *M*_P_(*T*) indicates that the thermal reduction rate of the surface moment and core moment are hardly distinguishable in the 6.5 nm Au-78 assembly. Apparently, the thermal reduction rate of the surface moment depends strongly on the particle size for small nanoparticles. A *b* = 1.1 is obtained for the 6.5 nm Au-78 assembly signals that the thermal reduction rate of core moment depends strongly on the particle size as well.

## 3. Experimental Section

### 3.1. Fabrication of Nanoparticle Powder

One way to fabricate small NPs is through nucleation in either physical or chemical processes. A couple of years before the turn of this century, Brust and co-workers developed a method based on two-phase reduction of gold salt that led to the successful synthesis of alkanethiol-functionalized Au NPs [50]. In this method, AuC1_4_^−^ is transferred from the aqueous solution to toluene using tetraoctylammonium bromide as the phase-transfer reagent and reduced with aqueous sodium borohydride in the presence of dodecanethiol C_12_H_25_SH [50]. Since then, enormous efforts have been made to obtain agent capped Au NPs using functional groups, such as amines [51], phosphine [52], alcohols [53], thiols [50], and even acids [23], that interact with surface Au atoms to prevent particle growth [54]. This method stabilizes Au NPs with a diameter as small as 2 nm, along with having the advantages of size being controllable and with a narrow size distribution. This method is nowadays immensely popular and useful.

NPs synthesized through chemical processes are unavoidably capped by some organic molecules or merged in organic matrices. These capping molecules can stabilize the NPs in the cores to prevent them from aggregating, but will also interact with them in ways that alter their basic properties. NPs free from capping agent are obviously needed for investigating the size effects on the NPs themselves. The gas condensation method employs a physical process involving the self-nucleation of atoms. The basic concepts behind this method include producing an atom vapor, nucleation through collisions, reducing the kinetic energies of the atoms, and cooling the NPs down to prevent interparticle fusion. Atom vapor can be produced through ohmic heating, by passing an electrical current through bulk ingots, until reaching their melting temperature. The kinetic energies of the evaporated atoms are reduced through collisions with surrounding inert gas atoms. Argon or helium gas is commonly used as the kinetic energy reducer. A collector maintained at a low temperature can prevent interparticle fusion. As a whole, the evaporated metal atoms coagulate to form nano-scale particles in the Ar/He atmosphere. The metal clusters are cooled through collisions with Ar/He atoms and are collected on a cold plate.

**Figure 14:**
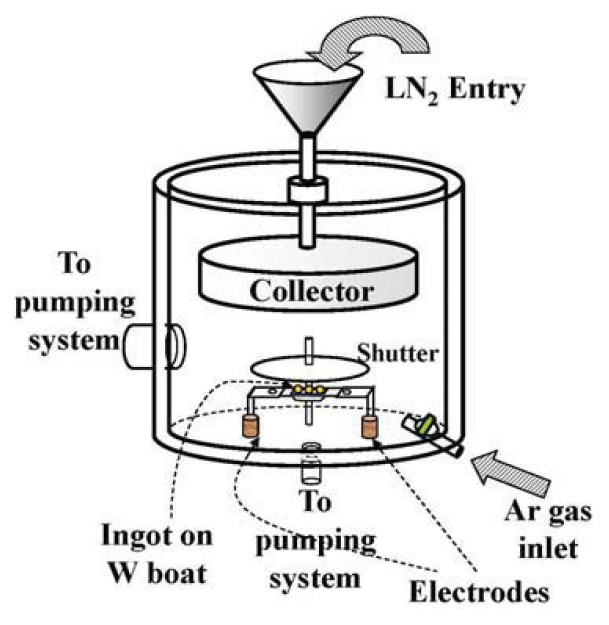
Schematic plot of the main body of the gas condensation chamber used for the fabrication of Au nanoparticles. It includes two electrodes providing an electric current through the bridging tungsten boat that supports the metal ingots, a non-magnetic stainless steel plate as the collector, a liquid nitrogen inlet on the back side of the collector, an Ar gas inlet and a vacuum pumping unit.

Figure 14 shows the main body of a gas condensation chamber. The main components include: two electrodes connected to a dc power supply, metal ingots placed on a tungsten boat that bridges the two electrodes, an Ar gas inlet to the chamber, a non-magnetic stainless steel plate for particle collection, a cryogen entry on the back side of the collector, and a vacuum pumping unit. There are several physical parameters that can be used to control the mean size and size distribution of the resultant nanoparticles: the heating electrical current through the metal ingots, the Ar pressure in the chamber during nucleation, the temperature of the collector, and the distance between the source and collector. The position of the collector is frequently fixed and the collector is cooled with liquid nitrogen. The cold collector acts as a cold trap for the NPs. A strong heating electrical current gives rise to a high evaporation rate that produces a size-dispersed powder. At a low evaporation rate, the key to control the mean particle diameter is the Ar pressure in the chamber during evaporation. This is the parameter that controls the times of collisions of the evaporated atoms before they reach the collector.

The Au NP powders used in this study are fabricated employing the gas condensation method. The mean particle size and size distribution are controlled by proper choices of chamber pressure and source temperature. High-purity Au spheres (~0.3 g, 99.99% pure and 2 mm in diameter) are heated by a current source (95 A) and are evaporated at a rate of 0.05 Å/s in an Ar atmosphere under various pressures (0.1 to 5 torr). The evaporated particles are collected on a non-magnetic SS316 stainless steel plate placed 20 cm above the evaporation source and maintained at 77 K. After restoration to room temperature, the NPs, which are only loosely attached to the collector, are stripped off. The samples thus fabricated are in powdered form and consist of macroscopic amounts of individual Au NPs. There is no substrate or capping molecules on the NPs. The resultant powders are no longer gold yellow but dark black, indicating that the absorption bands of the powders have blue shifted to the invisible region, as is the cases with most metallic NPs. It appears that the samples are quite stable against exposure to the air for a limited time, as the X-ray diffraction patterns and magnetization curves of the as-grown sample and the sample exposed to the air for 2 days are indistinguishable. However, the samples used in the present studies are kept in a vacuum at all times, except when being stripped off from the collector and loaded into the sample holders for magnetization measurements. These processes occur in an Ar atmosphere and take less than 2 min. For simplicity, the nanoparticle powder fabricated using a heating current of *b* A will hereafter be designed as Au-*b*.

### 3.2. Characterization of Nanoparticle Powder

It is very important to identify the basic structure of the fabricated samples. For NP assemblies, the crystalline structure, the chemical composition, the mean particle size, the size distribution and even the shape of individual particles, all have an essential effect on their magnetic properties. The commonly accessible means, such as X-ray diffraction (XRD), X-ray fluorescence (XRF), energy dispersive X-ray spectroscopy (EDXS) and atomic absorption spectroscopy (AAS) can be used to characterize the structure and purity of the assemblies; whereas the XRD peak profiles, atomic force microscopy (AFM) and transmission electron microscopy (TEM) images can reveal the size distribution and shape of individual particles.

XRD is frequently used to determine the crystalline structure and chemical composition, but is rarely used to extract the size distribution of the powder sample. It is, however, known that the width of a diffraction peak reflects the spatial extension of the lattice periodicity, which corresponds to the size of the nanoparticle. Diffraction peaks from a finite-sized particle are broader than the instrumental width. The Scherrer equation [55] is frequently used to determine the particle size. This equation, however, is formulated for a mono-sized system, and considers no size dispersion. The line profile of a diffraction peak from a size-dispersed powder can be obtained by combining the diffraction profiles contributed from each individual particle in the powder. The profiles of the diffraction peaks can be calculated once the size distribution of a powder sample is known. The line profile of a diffraction peak is very informative, and can be used to extract size distribution of a powder sample. AFM and TEM can image an individual NP with a sub-nanometer spatial resolution. It can be used to examine the sizes and shapes of NPs, making it feasible to obtain a reliable size distribution for a NP powder when it is properly sampled. NPs must be deposited very dispersedly on a flat holder for morphology mapping. Aggregated NPs frequently block the motion of the probe when scanning across the samples. The following process is used to avoid the aggregation among NPs for AFM image taking: gently shake the powder well, dissolve a very small amount of NPs into an appropriate amount of alcohol (<0.1 mg/cm^3^), vibrate the solution in an ultrasonic bath using a low frequency and power, let the solution stand still at least 24 h, gently extract a tiny drop from the middle section of the solution onto the flat holder, spin the holder to deposit dispersed NPs on the holder.

**Figure 15:**
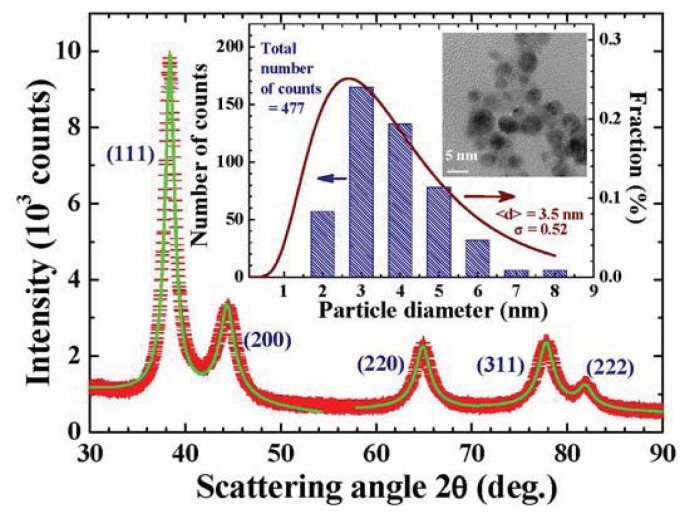
X-ray diffraction pattern of the Au-74 particle assembly at 300 K, revealing a face centered cubic crystalline structure. The solid curves indicate the calculated pattern assuming a log-normal size distribution with a center at 3.5 nm and a standard deviation width of 0.52. The inset shows the size distributions obtained from the X-ray diffraction profile (solid curve) and from AFM and TEM images (vertical bars).

Size analysis based on the AFM and TEM images reveals the size distributions of the Au NP powders used in the present studies. They can be described using log-normal functions. The X-ray diffraction patterns of all Au NP powders are associated with a face-centered cubic Au structure. As expected, the diffraction peaks appear to be much broader than the instrumental resolution, reflecting the finite-size effect. The mean particle diameters are determined by fitting the diffraction peaks to the diffraction profiles of finite sized particles, assuming a log-normal size distribution as is indicated by the AFM and TEM images. Figure 15 shows the observed (crosses) and fitted (solid curves) X-ray diffraction patterns of the representative Au-74 NP powder at 300 K, where the solid curves indicate the calculated pattern assuming a log-normal size distribution (solid curve in the inset to Figure 15) with a center at 3.5 nm and a standard deviation of 0.52. This size distribution obtained from the XRD peak profiles agrees well with that obtained from the AFM and TEM images (vertical bars) shown in the inset to Figure 15. Chemical analysis by means of XRF and AAS has also been performed on all Au NP powders to search for impurities. The XRF spectra are taken on a Rigaku ZSK Primus II spectrometer, employing the standard setup to scan through ~5 mg of the Au NPs. No fluorescence lines other than those from Au are detected. The AAS spectra are taken on a Perkin-Elmer AAnalyst 700 spectrometer, employing a graphite furnace to atomize ~3 mg of the Au nanoparticles with a cathode discharge lamp used for detecting Fe and Ni. No traces of oxidation phases or elements other than Au may be identified from the XRD/EDXS/XRF/AAS spectra. We estimate the impurity phases in the samples to be less than 20 parts per million. The chamber pressure and heating current employed in fabrication, the mean particle diameters, standard deviations of log-normal size distribution profile and lattice constants for the seven sets of NP powder used in the present study are all listed in Table 1.

## 4. Conclusions

The noble metal element Au in its bulk form is known to be weakly diamagnetic owing to its filled narrow *d*-band and a single half-filled free electron *s*-band. Many studies related to the magnetic properties of Au nanoparticles have been performed on polymer-capped Au nanoparticles. The present study, nevertheless, focuses on identifying the magnetic properties and the critical particle size for developing sizable spontaneous magnetic moments of bare Au nanoparticles. Seven sets of bare Au nanoparticle assemblies were fabricated employing the gas condensation method, which adopts a physical process involving the self-nucleation of atoms to form capping free Au nanoparticles. The resultant nanoparticles were no longer golden yellow but dark black, indicating that the absorption bands of the nanoparticles had blue shifted to the invisible region. The line profiles of X-ray diffraction peaks were used to determine the mean particle diameters and size distributions of the nanoparticle assemblies. Log-normal size distributions were assumed for the nanoparticle assemblies, which allowed us to calculate the diffraction profiles effectively and perform fits to the observed diffraction patterns.

The paramagnetic responses of the Au nanoparticle powders to a driving magnetic field were detected at all temperatures, showing the appearance of an extra magnetic component that overcomes the diamagnetic responses. The magnetization curves *M*(*H*_a_) reveal Langevin field profiles. Magnetic hysteresis were clearly revealed in the low field regime even at 300 K. Contributions from the different size particles in the nanoparticle assemblies to the magnetization were considered in analyzing the *M*(*H*_a_) curves, which allowed us to extract the dependency of particle moment on particle diameter μ*_p_*(*d*). It is found that the μ*_p_*(*d*) obtained from different Au nanoparticle assemblies match nicely, showing that the analysis is reasonably sound. The maximum particle moment is then predicted to appear in 2.4 nm Au particles, with a magnetic moment of 0.59 μ_B_ at 1.8 K when assuming the magnetic moments are evenly distributed in the nanoparticles. Magnetic field induced Zeeman magnetization from the quantum confined Kubo gap opening was also observed in Au nanoparticles smaller than 9.5 nm in diameter. Analyses of Zeeman magnetization allowed us to extract the value of the Kubo energy gap. The critical particle size for the development of spontaneous magnetic moment is identified through the size dependence of saturation magnetization *M*_P_. It was found that *M*_P_(*d*) departs from the 1/*d* dependence, but can be described by a log-normal function. The results indicate the maximum saturation magnetization will appear in 2.4 nm particles, which matches nicely with what is predicted for maximum particle moments. Magnetization will be hardly detectable for Au particles larger than 30 nm in diameter.

The general picture established so far for the magnetism in polymer-capped Au nanoparticles is that the surface atoms are ferromagnetic while the core atoms are Pauli paramagnetic. The coupling between the protective agent and the particle surface is a prerequisite for the development of spin polarized moments in Au nanoparticles. This creates localized 5*d* holes for ferromagnetism in the surface region. Ferromagnetism is thus associated only with the Au atoms on the surface while the core atoms remain diamagnetic. The present study shows that both the surface and core atoms contribute to the ferromagnetism. It is clear that the origins of the development of the magnetic moment in the present capping-free bare Au nanoparticles are fundamentally different. We believe that this is caused by charge redistribution in nano-sized particles triggered by charge transfer between the surface and core atoms matching the Fermi energies of the two regions and by the disruption of lattice periodicity at the surface. Although the moments developed in the Au nanoparticles are relatively weak, localized 5*d* holes do exist to reveal ferromagnetism. The paramagnetic behavior is associated with density of the 6*s* conduction electrons. The present observations involve both conduction 6*s* electrons and localized 5*d* holes. This can happen only when the 6*s* and 5*d* bands in Au nanoparticles are energetically close to each other.

## Figures and Tables

**Table 1:** The chamber pressure *P*, heating current *I*, lattice constant at room temperature *a*, mean particle diameter *d* and standard deviation σ of the log-normal size distribution of the seven sets of Au nanoparticle powders used in the present study. The nanoparticle powder fabricated using a heating current of b amp is labeled Au-b.

Label	*P* (torr)	*I* (amp)	*d* (nm)	σ	*a* (Å)
Au-74	3.0	74	3.5	0.52	4.0796(2)
Au-76	3.0	76	4.1	0.35	4.0795(5)
Au-78	3.2	78	6.5	0.40	4.0763(9)
Au-80	3.0	80	9.5	0.35	4.0762(9)
Au-82	2.5	82	13.4	0.35	4.0765(9)
Au-84	3.0	84	15.8	0.20	4.0782(7)
Au-86	3.5	86	17.5	0.35	4.0787
